# *In Vivo* Activity of MiR-34a Mimics Delivered by Stable Nucleic Acid Lipid Particles (SNALPs) against Multiple Myeloma

**DOI:** 10.1371/journal.pone.0090005

**Published:** 2014-02-27

**Authors:** Maria Teresa Di Martino, Virginia Campani, Gabriella Misso, Maria Eugenia Gallo Cantafio, Annamaria Gullà, Umberto Foresta, Pietro Hiram Guzzi, Maria Castellano, Anna Grimaldi, Vincenzo Gigantino, Renato Franco, Sara Lusa, Mario Cannataro, Pierosandro Tagliaferri, Giuseppe De Rosa, Pierfrancesco Tassone, Michele Caraglia

**Affiliations:** 1 Department of Experimental and Clinical Medicine, Magna Graecia University and Medical Oncology Unit, Catanzaro, Italy; 2 T. Campanella Cancer Center, “Salvatore Venuta” University Campus, Catanzaro, Italy; 3 Department of Pharmacy, Federico II University of Naples, Naples, Italy; 4 Department of Biochemistry, Biophysics and General Pathology, Second University of Naples, Naples, Italy; 5 Department of Medical and Surgical Sciences, Laboratory of Bioinformatics Unit, “Salvatore Venuta” University Campus, Catanzaro, Italy; 6 Pathology Unit, National Institute of Tumours of Naples “Pascale”, Naples, Italy; 7 Sbarro Institute for Cancer Research and Molecular Medicine, Center for Biotechnology, College of Science and Technology, Temple University, Philadelphia, Pennsylvania, United States of America; Federico II University of Naples, Italy

## Abstract

Multiple myeloma (MM) is a disease with an adverse outcome and new therapeutic strategies are urgently awaited. A rising body of evidence supports the notion that microRNAs (miRNAs), master regulators of eukaryotic gene expression, may exert anti-MM activity. Here, we evaluated the activity of synthetic miR-34a in MM cells. We found that transfection of miR-34a mimics in MM cells induces a significant change of gene expression with relevant effects on multiple signal transduction pathways. We detected early inactivation of pro-survival and proliferative kinases Erk-2 and Akt followed at later time points by caspase-6 and -3 activation and apoptosis induction. To improve the *in vivo* delivery, we encapsulated miR-34a mimics in stable nucleic acid lipid particles (SNALPs). We found that SNALPs miR-34a were highly efficient *in vitro* in inhibiting growth of MM cells. Then, we investigated the activity of the SNALPs miR-34a against MM xenografts in SCID mice. We observed significant tumor growth inhibition (p<0.05) which translated in mice survival benefits (p = 0.0047). Analysis of miR-34a and NOTCH1 expression in tumor retrieved from animal demonstrated efficient delivery and gene modulation induced by SNALPs miR-34a in the absence of systemic toxicity. We here therefore provide evidence that SNALPs miR-34a may represent a promising tool for miRNA-therapeutics in MM.

## Introduction

microRNA (miRNA) as therapeutics are an emerging area of investigation [1], [2]. miRNAs play a crucial role in regulation of gene expression [3] and may represent therefore powerful therapeutic agents. However, an important limitation for their use is linked to the unstable nature of the molecular structure [4], to the rapid plasma clearance and to their poor intracellular uptake that requires specific delivery strategies. Nanotechnology-based approaches have been recently used both to increase RNA stability *in vivo* and to enhance RNA uptake into tumor cells. In this light, the use of stealth nanocarriers allows the increase of RNA delivery in tissues characterized by increased vessel permeability and decreased lymphatic drainage, such as tumors [5]. Among the proposed nanocarriers, lipid-based vesicles, and in particular stable nucleic acid lipid particles (SNALPs) are characterized by high vesicle loading, good transfection efficiency and stability in serum [6]. SNALPs have been successfully proposed to deliver small interfering RNAs in non-human primates [7] and clinical trials are currently ongoing. Based upon these considerations, SNALPs appears an interesting developmental approach to deliver miRNAs in tumors.

miR-34a belongs to a miRNA family that includes also miR-34b and miR-34c and was firstly found to be a tumour suppressor (TS) miRNA [8]. The tumor suppressor TP53 induces miR-34a transcription and this effect is paralleled by apoptosis, cell-cycle arrest, and senescence [9]–[14]. The mutation of p53 with the consequent loss of function can be functionally counteracted by the addition of miR-34a in pancreatic cancer cells [15], [16]. However, it was also recently found that miR-34a activity can be independent from TP53 mutational status in different cell systems [17], [18]. In addition, the activity of miR-34a is not limited to miR-34a defective cell lines [18].

Multiple myeloma (MM) is a hematologic malignancy, which needs development of novel therapeutic strategies [19]. Deregulated expression of miRNAs in MM cells has been widely demonstrated [20], thus eliciting interest for these molecules also as antitumor therapeutic agents [21]–[26]. In this light, we previously reported that lipidic-formulated miR-34a has anti-MM activity *in vivo* in SCID mice bearing human MM cells [27]. Moreover, we recently demonstrated that SNALPs can be successfully used to deliver miR-34a in an in vitro model of medulloblastoma [28], but the *in vivo* delivery of miR-34a in SNALPs warrants still additional investigations.

Here, we investigated SNALPs as effective agents to deliver miR-34a *in vivo*. In details, the study was carried out in an experimental model of MM taking into account that efficient miR-34a delivery could be the basis for new therapeutic strategies for this disease [19], [29]. Firstly we generated and characterized miR-34a encapsulating SNALPs. Then, we characterized the effects of miR-34a on signal transduction pathways involved in regulation of both proliferation and apoptosis in MM cells. Finally, we studied the effect of the SNALP miR-34a formulation on tumor growth and mice survival and *in vivo* effects in MM tissues.

## Materials and Methods

### Materials

1,2-dioleyl-3-dimethylammonium propane (DODAP) and N-palmitoyl-sphingosine-1-succinyl[methoxy(polyethylene glycol)2000] (PEG2000-Cer16) were purchased by Avanti Polar Lipids. Disteroylphosphatidylcholine (DSPC) was kindly offered from Lipoid GmbH (Cam, Switzerland). Cholesterol (CHOL), sodium chloride, sodium phosphate, HEPES, citric acid and sodium citrate was purchased by Sigma Aldrich (USA), ethanol and other reagents were obtained by Carlo Erba Reagenti (Italy). miR-34a were purchased by Life Technologies as ds-oligonucleotide with the sequence of miR-34a duplex as reported in miR.org database. As control an oligonucleotide with scrambled sequence (miR-NC) was used (Life Technologies).

### Preparation of Stable Nucleic Acid Lipid Particles (SNALPs)

SNALPs formulations were prepared by modified ethanol injection method. Briefly, lipid stock solutions were prepared in ethanol; determined amounts were transferred in a glass tube to obtain a 0.4 ml lipid mix with the following composition: DSPC/CHOL/DODAP/PEG2000-Cer16 (molar ratio 25/45/20/10). In a separated tube, 0.2 mg of miR-34a were dissolved in 0.6 ml of 20 mM citric acid at pH 4.0. The two solutions were warmed for 2–3 min to 65°C and the lipid solution were quickly added to the miRNA solution under stirring. The mixture was passed 5 times through 200 nm and 20 times through 100 nm polycarbonate filters using a thermobarrel extruder (Northern Lipids Inc., Vancouver, BC, Canada) maintained at approximately 65°C. Therefore, the preparation was dialyzed (3,5 kDa cutoff) against 20 mM citrate buffer at pH 4.0 for approximately 1 h to remove excess of ethanol, followed by further dialysis against HBS (20 mM HEPES, 145 mM NaCl, pH 7.4) for 12–18 h to remove the citrate buffer and to neutralize the DODAP. Not encapsulated miRNA was removed by DEAE-Sepharose CL-6B column chromatography. The formulation was prepared in triplicate.

### SNALPs Characterization: Mean Diameter, Polydispersity Index and Zeta Potential

The mean diameter and size distribution of SNALPs were determinate at 20°C by photon correlation spectroscopy (PCS) (N5, Beckman Coulter, Miami, USA). Each sample was diluted in deionized/filtered (0.22 ìm pore size, polycarbonate filters, MF-Millipore, Microglass Heim, Italy) water and analyzed with detector at 90° angle. As measure of the particle size distribution, polydispersity index (PI) was used. For each batch, mean diameter and size distribution were the mean of three measures. For each formulation, the mean diameter and PI were calculated as the mean of three different batches. The zeta potential (ZP) of the SNALPs was determined in distilled water at 20°C by Zetasizer Nano Z (Malvern, UK). For each batch, mean diameter, size distribution and ZP were the mean of three measures.

### miR-34a Encapsulation into SNALPs Formulations

The amount of miRNA encapsulated into the SNALPs was measured spectrophotometrically. Briefly, 10 ìl of SNALPs suspension were dissolved in 990 ìl of methanol and analysed at 260 nm. Actual loading was calculated as amount (mg) of miRNA/mg of mg total lipids. The amount of miRNA loaded into the nanocarriers was expressed as miRNA actual loading and encapsulation efficiency, calculated as mg of miRNA/mg of total lipids and percent ratio between miRNA actually loaded into SNALPs and miRNA theoretical loading, respectively. For each batch, miRNA loading was the mean of three measures. For each formulation, the miRNA loading was calculated as the mean of the measures obtained in three different batches (n = 3). The phospholipid content of the carrier suspension was determined by the Stewart assay [30]. Briefly, an aliquot of the SNALP suspension was added to a two-phase system, consisting of an aqueous ammonium ferrithiocyanate solution (0.1 N) and chloroform. The concentration of DSPC was obtained by measure of the absorbance at 485 nm into the organic layer.

### MM Cell Lines

SKMM-1 MM cell lines were available within our research network. Cells were grown in RPMI medium, containing L-glutamine (Gibco®, Life Technologies, Carlsbad, CA), with the addition of 10% fetal bovine serum (Lonza Group Ltd., Switzerland), 100 U/ml penicillin, and 100 mg/ml streptomycin (Gibco®, Life Technologies) at 37°C in a 5% CO_2_ atmosphere.

### Gene-expression Profiling

Gene expression profiles were obtained from SKMM-1 cells after transfection with miR-34a or NC in 3 parallel experiments. 24 hours after transfection cells were collected and used for total RNA (tRNA) extraction by Trizol lysis buffer and column purification with RNeasy kit (Qiagen, Hilden, Germany). A total of 300 ng RNA were used as starting material for preparing the hybridization target by using the Ambion® WT Expression Kit (Ambion, Life Techologies). The integrity, quality and quantity of tRNA were assessed by the Agilent Bioanalyzer 2100 (Agilent Technologies, Santa Clara, CA) and NanoDrop 1000 Spectrophotometer (Thermo Scientific, Wilmington, DE). The amplification of cRNA, the clean up and the fragmentation were performed according to the Affymetrix’s procedures. Microarray data were generated by Human GeneChip 1.0 ST (Affymetrix Inc., Santa Clara, Ca) containing 764,885 distinct probes that interrogate 28,869 well-annotated genes. Arrays were scanned with an Affymetrix GeneChip Scanner 3000. Raw data produced by the Affymetrix Platform (i.e. CEL files) were processed using Affymetrix Expression Console (EC). Pre-processing phase including normalization and annotation of data was performed according to Affymetrix guidelines and micro-CS software as previously described by us [25]. Clustering and fold change (FC) analysis were done using the dChip software [31], and biological pathways modulation by miR-34a were performed by Ingenuity Pathway Analysis (IPA®) platform (Ingenuity System, Redwood city, CA) as previously reported [25].

### Western Blot Analysis

SKMM1 MM cells were transfected with miR34a as previously described [27]. For cell extract preparation, cells were washed twice with ice-cold PBS/BSA, scraped and centrifuged for 30 min at 4°C in 1 ml of lysis buffer (1% Triton, 0.5% sodium deoxycholate, 0.1 M NaCl, 1 mM EDTA, pH 7.5, 10 mM Na_2_HPO_4_, pH 7.4, 10 mM PMSF, 25 mM benzamidin, 1 mM leupeptin, 0.025 U/ml aprotinin). Equal amounts of cell proteins were separated by SDS-PAGE. The proteins on the gels were electro-transferred to nitrocellulose and reacted with the different MAbs. Rabbit antisera raised against Erk-1/2, and pErk MAb were purchased from Santa Cruz Biotechnology (Santa Cruz, CA). Rabbit antisera raised against pGSK3 α/β, Akt and the relative activity evaluation kit were purchased by Cell Signalling (Cell Signaling Technology, Beverly, MA). Anti-pro-caspase-3 and pro-caspase-6 MAbs were purchased from Alexis (Lausen, Switzerland). Anti-α-tubulin MAb was purchased from Oncogene (Cambridge, MA).

### *In vitro* Analysis of SNALP miR-34a Formulations

For cell proliferation analysis, 1.5×10^5^ MM cells were plated in 6 well plates, and cultured in presence of 100 nM of different SNALP miR-34a formulations, and then harvested and counted at 24-hour intervals using a Trypan Blue-excluding viable cells assay. Each sample was run in triplicate and the experimental procedure was repeated in four independent experiments.

### *In vitro* Apoptotic Analysis by TUNEL Assay

The apoptotic cell rate was assessed by the TUNEL assay (In Situ Cell Death Detection Kit, TMR red; Roche Applied Science, Basel, Switzerland). The SKMM-1 cells were seeded and transfected with miR-34a or NC as described above. After 12, 24, 48 or 72 hours from transfection, 5×10^5^ cells were washed with PBS and fixed with 4% paraformaldehyde in PBS (pH 7.4) at room temperature for 1 hour and then suspended in 0.1% sodium citrate containing 0.1% Triton X-100 for 2 minutes on ice. Cells were first treated with TUNEL reaction mixture containing terminal deoxynucleotidyl transferase (TdT) and fluorescein-dUTP, and then incubated at 37°C in a humidified atmosphere in the dark for 1 hour according to the manufacturer’s instructions. The TdT catalyzes the binding of fluorescein-dUTP to free 3′-OH ends in the nicked DNA. After washing with PBS, the cells were analyzed with a flow cytometer (FACScan; BD Biosciences) equipped with a 540-nm excitation laser. Data analysis was performed with the specific software (Cell Quest). Results were shown as percentages of red fluorescence-emitting SKMM-1 cells (apoptotic cells).

### SNALP miR-34a Activity in *in vivo* Models of Human MM

Male CB-17 severe combined immunodeficient (SCID) mice (6- to 8-weeks old; Harlan Laboratories, Inc., Indianapolis) were housed and monitored in our Animal Research Facility. All experimental procedures and protocols had been approved by the Institutional Ethical Committee (*Magna Graecia* University) and conducted according to protocols approved by the National Directorate of Veterinary Services (Italy) (Permit Number: 235 on 30^th^ June 2011). In accordance with institutional guidelines, mice were sacrificed when their tumors reached 2 cm in diameter or in the event of paralysis or major compromise in their quality of life, to prevent unnecessary suffering. For our study 15 SCID mice were inoculated in the interscapular area (sc) with 5×10^6^ MM cells in 100 µL RPMI-1640 medium [32]. After detection of palpable tumors, approximately 3 weeks following injection of MM cells, animals were randomized into 3 groups including 5 mice per group, that received the following treatments: *i*) SNALP empty *ii*) SNALP miR-NC *iii*) SNALP miR-34a. Each animal received a dose of 20 µg of miR-34a. The treatment schedule included 5 treatments, three days apart, via tail vein. The tumor sizes were measured every two days until the day of first mouse sacrifice, using a caliper, and volume was calculated using the formula: V = 0.5×a×b^2^, where a and b are the long and short diameter of the tumor, respectively. The survival time was defined as the time interval between the start of the experiment and either death or the day of mouse sacrifice. Tumors and vital organs including liver, kidney and heart were collected and placed in either 10% formalin for histology or in RNA*later*® for RNA isolation.

### Quantitative Real-time PCR of miR-34a and NOTCH1 mRNA

Total RNA (tRNA) including low molecular weight RNA was isolated from xenografts at the end of treatment schedule, by TRIzol® Reagent (Invitrogen, Life Technologies, Carlsbad, CA, USA) according to manufacturer’s instructions. Tissues disruption was performed using a TissueRuptor® (Qiagen, Venlo, Netherlands) according to manufacturer’s instructions. The single-tube TaqMan miRNA assays (Applied Biosystems, Life Technologies) was used to detect and quantify mature miR-34a (assay ID 000426), by the use of ViiA7 detection system (Applied Biosystems, Life Technologies). miRNAs expression was normalized on RNU44 (assay ID 001094) housekeeping (Applied Biosystems). For NOTCH1 mRNA quantification, Oligo-dT-primed cDNA was obtained using the High Capacity cDNA Reverse Transcription Kit (Applied Biosystems), then used to quantify mRNA levels by Taqman assay (assay ID Hs01062014_m1). Normalization was performed with GAPDH (assay ID Hs03929097_g1, Applied Biosystems). Comparative real-time polymerase chain reaction (RT-PCR) was performed in triplicate, including no-template controls. Relative expression was calculated using the comparative cross threshold (Ct) method [33].

### Histology and Immunohistochemistry

At the end of observation, animals were sacrificed and livers, kidneys and tumors were retrieved. Tumors were immediately immersed in 4% buffered formaldehyde and after 24 h, washed, dehydrated, and finally embedded in paraffin. Haematoxylin-eosin staining was performed using 4 µm tumors section which were mounted on poly-lysine slides. Immunohistochemical staining has been done on slides from formalin-fixed, paraffin embedded tissues, to evaluate Phospho-Akt expression in myeloma cells xenografted in mice. Phospho-Akt [(Ser473) (736E11) Rabbit mAb] antibodies from Cell Signaling Technology (Danvers, MA) were used to stain mice myeloma. Samples were processed with peroxidase detection system reagent kit (Novocastra, Wetzlar, Germany). Apoptosis was evaluated by the terminal deoxynucleotidyl transferase (TdT)-mediated dNTP-labeling (TUNEL) method using Fragel DNA fragmentation detection kit colorimetric-TdT enzyme by Calbiochem–Merck KgaA (Darmstadt, DK). Four µm-thick sections were deparaffinized and rehydrated and antigen retrieve technique was carried out in pH 6.0 buffer in a microwave for 3 minutes using standard histological technique. Evaluation has been done by two expert pathologists (RF and VG) and interpreted using a light microscope (Olympus, NY).

### Statistical Analysis

Student’s *t* test, two-tailed, and Log rank test were used to calculate all reported *P*-values using GraphPad software (www.graphpad.com), with minimal level of significance specified as *P*<0.05. Graphs were obtained using Microsoft Excel tool.

## Results

### miR-34a Induces Perturbation on Whole MM Cell Transcriptome

**Figure 1:**
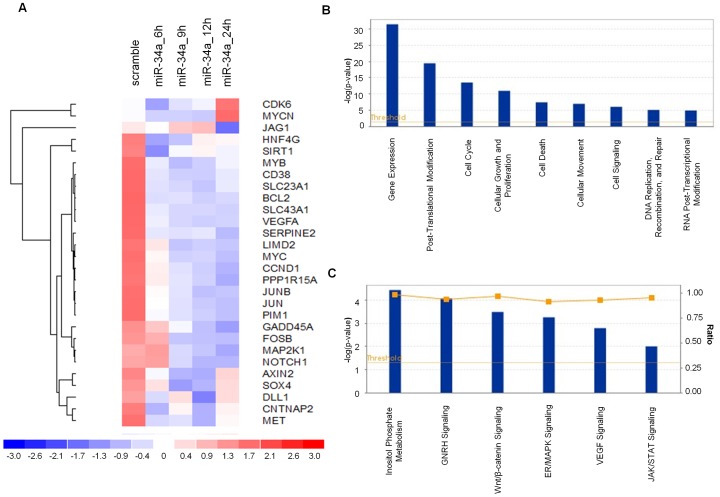
Whole Gene profiling perturbations induced by synthetic miR-34a. A) Heatmap representation of the top 28 down- and up-regulated genes (P<0.001) following miR-34a or miR-NC transfection in SKMM-1 cells by Gene 1.0 ST array chip (Affymetrix) and DChip software. Data are presented row normalized (range from −3 to +3 standard deviations from median in expression). Genes that underwent a 1.5-fold change as compared to control, were selected and clustered. Assays performed in triplicate are shown. Ingenuity Pathway analysis of biological function annotation B) and canonical pathways C) for differential expressed gene (FC = +1.5) after miR-34a transfection respect to the miR-NC control. The bar graphs show pathways most modulated by miR-34a inhibitors as compared to control, based on statistical significance (P-value and ratio). The yellow line indicates the threshold of significance.

To investigate the molecular bases of miR-34a tumor inhibition in MM, we evaluated the effects of miR-34a at the trascriptome level by performing gene expression analysis in SKMM-1 cells transfected with synthetic miR-34a or miR-NC in a time course experiment. Following electroporation of cells, the tRNA was isolated for gene expression profiling and analyzed by Affymetrix Human GeneChip 1.0 ST. After running Plier summarization and quantile normalization algorithms, we performed a class comparison analysis of miR-34a transfected cells *versus* control considering the whole gene profile for each time point. Unsupervised hierarchical clustering segregated samples based on treatment assignment, suggesting a common transcriptional consequence in response to miR-34a transfection. These perturbations did not prompt global, non specific silencing but instead produced significant changes in a finite number of genes that mostly occurred at 9 and 12 hours after miR-34a transfection. Then, we selected and analyzed the profiles of modulated genes known to be target of miR-34a as stored in TargetScan database [34] obtaining a list of 475 targets. Figure 1 A shows the heatmap representation of the top 28 down- and up-regulated genes following miR-34a transfection in the experimental time series analysis. As evidenced by functional enrichment analysis performed using DAVID [35] top enriched terms included “regulation of cell proliferation” and “cell cycle” as well as “regulation of transcription DNA-dependent” and “regulation of transcription from RNA polymerase II promoter” (p-value<0.05 after Bonferroni correction). To analyze higher-order influences on biological networks regulated by miR-34a, gene data sets underwent Ingenuity Pathway Analysis (IPA®). As shown in figure 1 B at both time points of 9 and 12 hours after miR-34a transfection, “cell death”, “cell cycle”, and “gene expression” were the most modulated biological function based on –log (p-value) score. Moreover, based on ratio (miR-34a/control) p53 signaling, CDK5 signaling as well as chemokine signaling pathways were the most modulated by miR-34a transfection in MM cells. (Figure 1 C). Therefore, the transfection of MM cells with miR-34a induces perturbation of cell death/proliferation pathways. On the basis of these data, we undergone evaluation of ERK and Akt-dependent pathways which have specific relevance in MM pathobiology [36], [37].

### miR-34a Inhibits Major Survival Pathways and Activates Caspase-dependent Apoptosis

**Figure 2:**
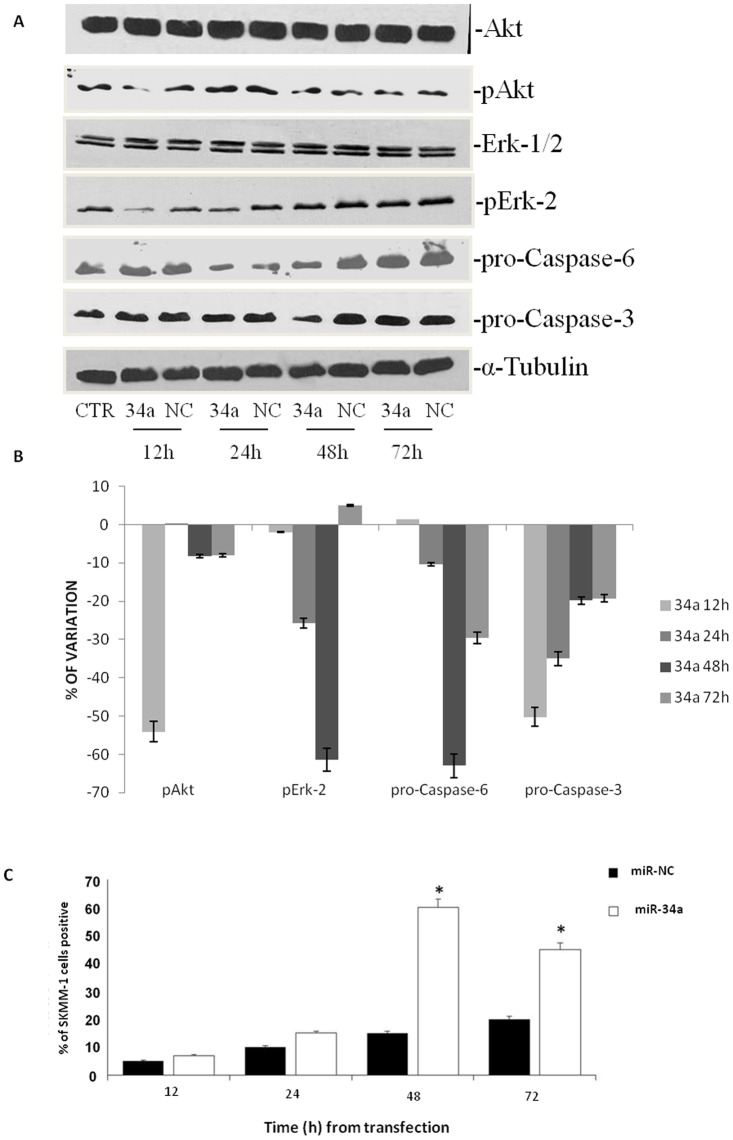
Effects of miR-34a replacement on survival pathways and apoptosis occurrence. A) SKMM-1 cells were transfected with miR-34a (34a) or scramble miR-NC (NC) and after different times from the transfection were collected for Western blot analysis. Thereafter, the expression and phosphorylation of Erk, the activity and expression of Akt and pro-caspase-6 and -3 expression were evaluated after blotting with specific antibodies, as described in “Material and Methods”. The house-keeping protein α-tubulin was used as loading control. Each point is representative of 3 different evaluations performed in 3 different experiments. B) Scan of the bands associated with pErk-2 expression and Akt activity normalized for total Erk-2 or Akt expression, respectively, and of pro-caspase-3 and pro-caspase-6 expression, normalized with the housekeeping protein α-tubulin in SKMM-1 cells, was performed with ImageJ software. The intensities of the bands were expressed as % of changes based upon determination of arbitrary units (%, mean of three different experiments). Each point is the mean of 3 different evaluations performed in at least 3 different experiments. Bars, s.e.’s. C) SKMM-1 cells after transfection with miR-34a (34a) or scramble miR-NC (NC). The cells were collected after the indicated times from the transfection and apoptosis was evaluated with TUNEL assay by FACScan as described in “Materials and Methods”. Results are shown as percentage of apoptotic cells. Data are the average ‘SD of 3 independent experiments.

We found that the transfection of MM cells with miR-34a induces a decrease of the phosphorylation of Erk-2 and of Akt activity as shown in Figure 2 A. In details, down-modulation of the two kinases was time-dependent reaching a peak (about 60% of decrease) after 12 h from the transfection (Figure 2 B). At later time points, the phosphorylation of Erk resembled miR-NC transfected cells while Akt activity was still reduced but at smaller extents (about 40% after 24 h and about 20% at 48 and 72 h, respectively) (Figure 2 B). In the light of pro-apoptotic signal transduction pathway modulation induced by miR-34a transfection, we evaluated apoptosis activation by the expression of the full length isoforms of the terminal caspases-3 and -6 and we found that miR-34a transfection induced a time-dependent cleavage of both enzymes (Figure 2 A). In details, the decrease of the full length caspasese-3 and -6 was detected already at 24 h after transfection (about 30% and 16%, respectively) and it became maximal 48 h after transfection (about 60% decrease for both) (Figure 2 B). Full length caspase-3 resembled miR-NC transfected cells after 72 h from the transfection while full length caspase-6 was still about 30% reduced at the same time point (Figure 2 B). We have also evaluated the activation of an apoptotic process in these cells through the evaluation of TUNEL at FACS analysis. We found maximal activation of apoptosis in miR-34a-transfected cells at 48 h, (35% apoptotic cells, Figure 2 C); at 72 h the apoptosis was recorded in about 55% cells. These data indicate that miR-34a transfection induced a strong decrease of the activation status of anti-apoptotic proteins Akt and Erk that was followed by cleavage of terminal caspases-3 and -6 and apoptosis induction.

### SNALPs Encapsulation of miR-34a Mimics

SNALPs encapsulating miR-34a (SNALP miR-34a) were prepared and characterized. SNALP miR-34a had a mean diameter of 157.2±17.2 and were characterized with a narrow size distribution (PI of about 0.16±0.03) and a negative ZP (−13.52±2.28). We prepared SNALPs with a theoretical loading of 200 µg ON/mg lipids and an actual loading of about 160 µg ON/mg lipids, corresponding to an encapsulation efficiency of about 82%.

### *In vitro* and *in vivo* Experiments

**Figure 3:**
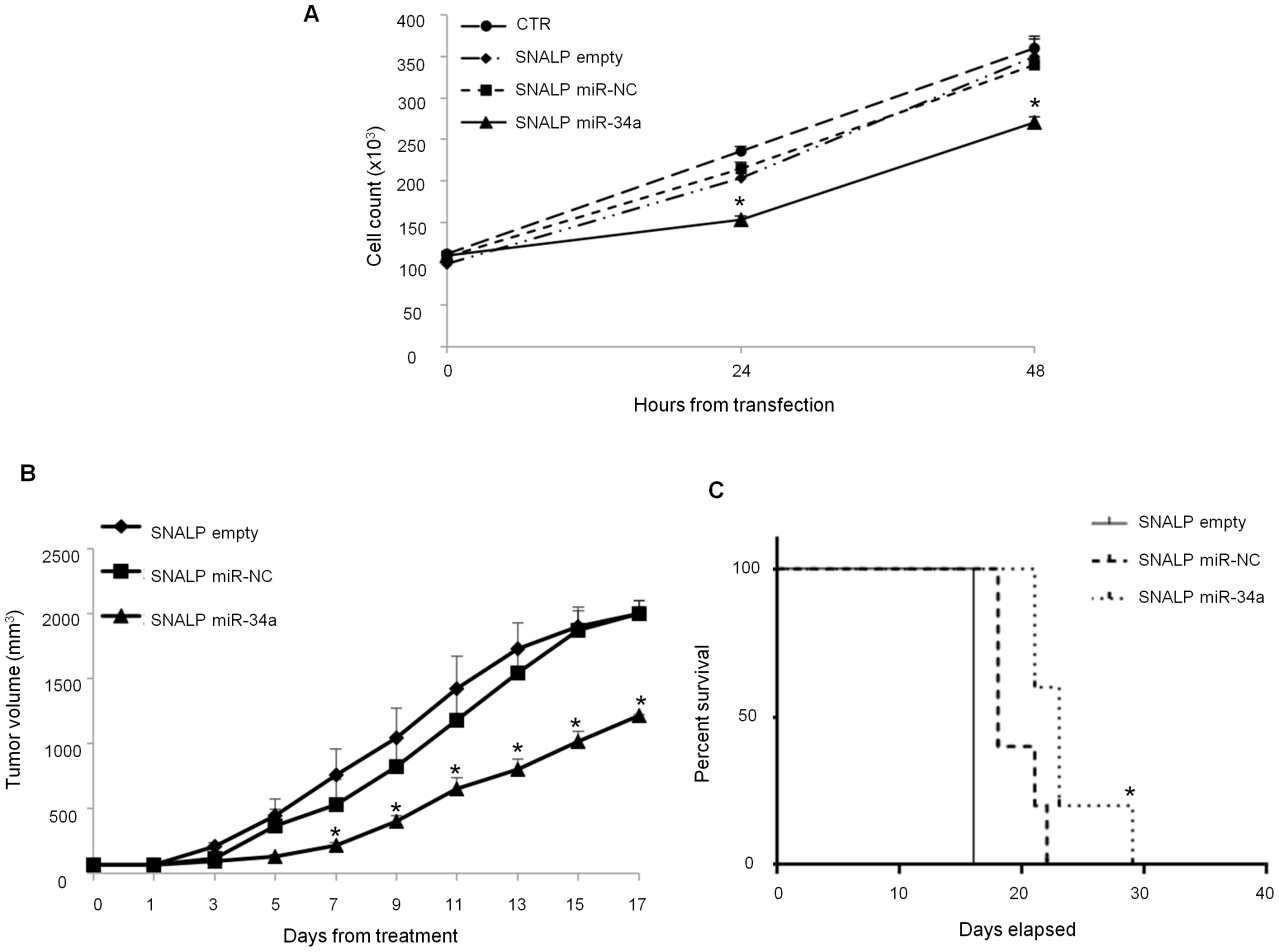
SNALPs formulated miR-34a has anti-proliferative activity against MM *in vitro* and *in vivo*. A) Trypan blue exclusion assay of SKMM-1 cells treated with SNALP-encapsulated miR-34a or scramble oligonucleotides as control (NC). Analysis was performed by microscope Burker chamber counts and trypan blue exclusion assay. Averaged values of three independent experiments are plotted including ±SD. P-values calculated by Student’s *t* test, two-tailed, at 24 and 48 hours after transfection, are respectively: 0.001 and 0.02 versus SNALP empty or 0.0099 and 0.01 versus SNALP miR-NC. B) Mice carrying palpable subcutaneous SKMM-1 tumor xenografts were treated by intravenous tail vein injections with 20 µg for each treatment of miR-34a encapsulated into SNALPs. As control SNALPs incapsulating scramble miR-NC or empty were used. Caliper measurement of tumors were taken every 2 days from the day of the enrollment. Averaged tumor volumes of 4 mice per group are reported±SD. (*) indicate significant *P*-values (*P*<0.05). D) Survival curves (Kaplan-Meier) of treated mice show prolongation of survival after SNALP formulated miR-34a treatment compared to controls (log-rank test, *P* = 0.0047 and 0.002 SNALP miR-34a *vs* empty and miR-NC, respectively). Survival was evaluated from the first day of treatment until death or sacrifice.

**Figure 4:**
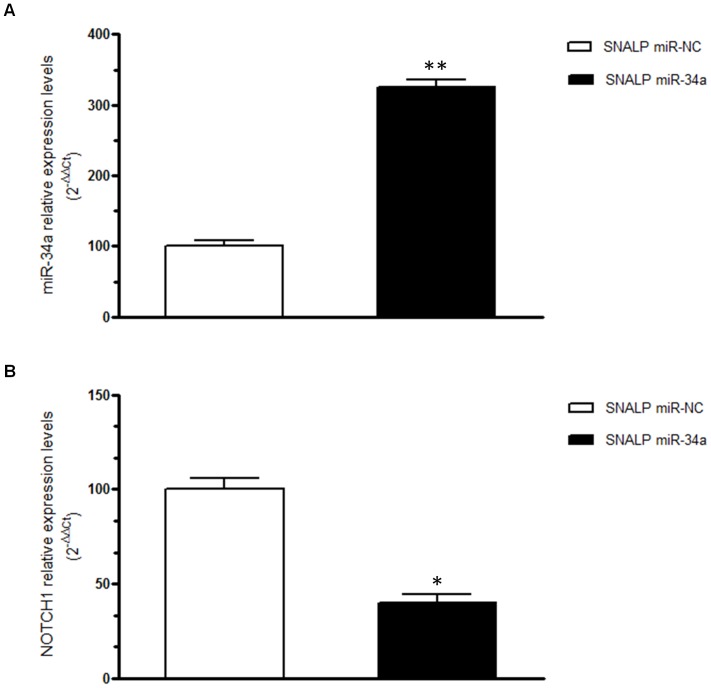
Effects induced by systemic delivery of miR-34a in MM xenografts. miR-34a q-RT-PCR A) and q-RT-PCR of NOTCH1 mRNA expression B) at the end of observation of animal treatments with SNALP miR-34a formulation and SNALP miR-NC as control, in retrieved xenograft SKMM-1 tumors. The results are shown as average of miR-34a or NOTCH1 mRNA expression level after normalization with RNU44 or GAPDH, respectively, and ΔΔCt calculations. Data represent the average of 3 independent experiments ±SD. (*) P<0.05, (**) P<0.01.

To confirm the biological activity of miR-34a formulated in SNALPs, we performed cell viability analysis by trypan blue exclusion assay. Cells were plated and treated with SNALPs encapsulating 100 nM of miR-34a or miR-NC, or empty SNALPs, or saline as control. Cell viability assay was performed at 24 and 48 hours after the beginning of the treatment. As shown in Figure 3 A, a significant inhibition of cell growth was observed after treatment with SNALPs encapsulating miR-34a if compared to empty SNALP after 24 and 48 hours of treatment (P = 0.001 and 0.02, respectively) or SNALP encapsulating miR-NC (P = 0.0099 and 0.01, respectively), reaching 50% of growth inhibition after 48 h of treatment. We next explored the effects of the *in vivo* systemic delivery of the miR-34a formulated in SNALPs in antagonizing the growth of MM xenografts. When sc MM tumors became palpable, mice were randomized and systemically treated, *via* tail vein, with either miR-34a or miR-NC encapsulating SNALPs at the same dose of 1 mg/kg per mouse or empty SNALPs. Following 5 injections (3 days apart), a significant anti-tumor effect of SNALP miR-34a formulation was detected (Figure 3B). Moreover, we observed 60% tumor growth inhibition (p<0.05), in mice treated with SNALP miR-34a after 17 days from the beginning of treatment if compared to the effects induced by empty SNALPs or SNALP miR-NC. The treatment with SNALP miR-34a induced a significant survival benefit in treated mice (p = 0.0047) (Figure 3 C). We have also evaluated the SNALP miR-34a delivery in tumor tissues and the modulation of its canonic target NOTCH1 (Figure 4 A and B, respectively). As expected, we found miR-34a enrichment and NOTCH1-mRNA downregulation in tumors treated with SNALP miR-34a as compared to controls (Figure 4 A and B, respectively). Finally, no mice weight reduction was observed in all animal groups (data not shown).

### SNALP miR-34a Reduces Akt Activation and Induces Apoptosis in MM Tissues in the Absence of Systemic Toxicity

**Figure 5:**
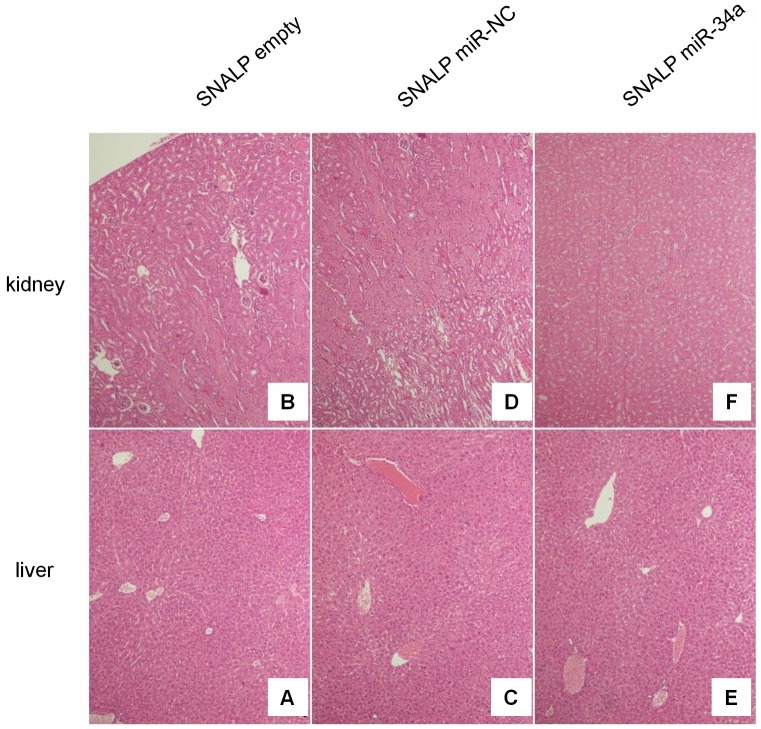
H&E staining of livers and kidney indicates absence of systemic toxicity. Hematoxylin and eosin staining (40-fold magnification) of kidney and liver retrieved from SNALP empty (A, B), SNALP miR-NC (C, D) and SNALP miR-34a (E, F) treated mice, respectively. No significant damage was detected in the different groups of treatment. Representative image are shown.

In order to assess the toxicity of miR-34a containing SNALPs, we collected livers and kidneys from animals at the time of sacrifice and tissues where analyzed by conventional hematoxylin/eosin staining. Normal histologic architecture of livers and kidney in all the examined animal groups was observed, in the absence of necrotic or other cell death events as shown in figure 5. Therefore, we can exclude toxic effects of SNALPs in our experimental model.

**Figure 6:**
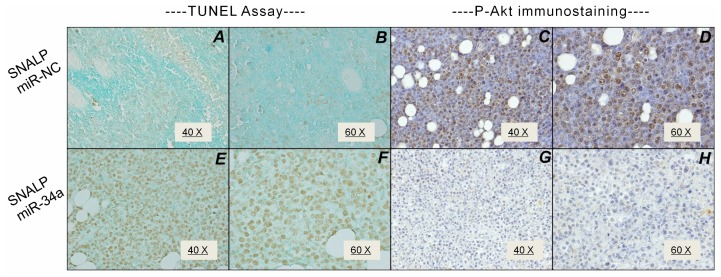
SNALP miR-34a reduces Akt activation and induces apoptosis in MM *in vivo*. TUNEL assay of SKMM-1 xenograft retrieved from SNALP miR-NC (A, B) and SNALP miR-34a (E, F) treated mice. The TUNEL positive cells are colored in brown. Representative image at 40-fold (A, E) and 60-fold (B, F) magnification are shown. p-Akt immunostaining SKMM-1 xenograft retrieved from SNALP miR-NC (C, D) and SNALP miR-34a (G, H) treated mice. Representative image at 40-fold (C, G) and 60-fold (D, H) magnification are shown.

We retrieved MM tumors and apoptosis was evaluated by TUNEL analysis. While the SNALP miR-NC did not induce significant apoptotic effects (Fig. 6A), SNALP miR-34a induced an about 50% apoptosis without evidence of necrosis (Fig. 6C). In these samples, we also evaluated the expression of pAKT. We detected 50% pAkt positive cells in SNALP miR-NC-treated tumors (Fig. 6B), while SNALP miR-34a strongly reduced pAkt expression that remained detectable in 20% of cells only (Fig. 6D). We have randomly evaluated apoptosis occurrence in normal tissues collected from mice and we have not found any increase in apoptotic index as assessed with TUNEL. Therefore, the administration of SNALP miR-34a induces anti-MM effects and signaling changes resembling *in vitro* findings and indicating a successful delivery of active miR-34a mimics in MM tumors.

## Discussion

Despite the recent development of novel preclinical platforms [38]–[41] and innovative drugs [42], MM is still an incurable disease. Recent findings highlighted miRNA therapeutics as an attractive option for the treatment of MM [29], [43], [44]. The development of miRNA replacement strategies is based upon the tumour suppressive activity of some of the known miRNAs. In this light, miR-34a belongs to a miRNA family that was firstly found to exert TS activity [8]. Its transcription is regulated by the TP53 protein. We recently reported a strong anti-tumour activity of miR-34a replacement strategies in different *in vivo* experimental models of MM [27]. We here explored the molecular effects induced by miR-34a on MM cell line (SKMM-1) expressing intermediate levels of miR-34a and carrying a mutated TP53. Indeed, we found that the transfection of these cells with miR-34a mimics induced a time-dependent expression modulation of 28 genes and IPA® analysis revealed the modulation of several signalling pathways involved in the control of cell proliferation and apoptosis. One of the most affected was the Erk/Akt-dependent pathway. These results are not surprising since it was recently demonstrated that the replacement of miR-34a in erithroleukemic K562 and colon cancer HCT116 cells causes deep modulation of gene expression including some of the genes that we found modulated in our *in vitro* model [45]. Moreover, the same authors described that miR-34a mimics are able to reduce activation of both Erk and Akt, providing confirmation to our findings from IPA analysis. In fact, we predicted perturbation of several pathways induced by miR-34a mimics transfection, some of them overlapping those described by Lal et al. [45]: i.e. Wnt/β-catenin signalling, Erk/MAPK signaling and VEGF signalling. miR-34a was also described to be involved in the negative regulation of the receptor tyrosine kinase AXL expression and of Akt activation in triple receptor negative breast cancer cells (MDA-MB-231) [46]. To our knowledge, we firstly demonstrated that miR-34a can induce sequential down modulation of both Erk and Akt activity, which is followed by pro-caspase-6 and -3 cleavage and apoptosis induction in MM cells. Based upon the high anti-proliferative activity of miR-34a mimics in MM, we investigated a nanotechnology-based delivery system to overcome the biopharmaceutical issues related to the administration of nucleic acids. Specifically, we used SNALPs that, unlike the cationic liposomes, are stable in serum and are characterized by high encapsulation and efficient transfection [6]. Results from ongoing clinical trials in other disease support our proposal that this delivery system could be a new therapeutical approach for MM by the use of miR-34a mimics. The analysis of the antiproliferative effects of SNALP miR-34a revealed efficient inhibition of SKMM-1 cell growth. An important key point of our work is the efficient systemic delivery of miR-34a mimics in MM xenografts in SCID mice. In fact, *in vivo* results were in agreement with *in vitro* experiments demonstrating the anti-MM activity of miR-34a encapsulated into SNALPs. It is possible to hypothesize that SNALPs work not only by enforcing the intracellular delivery of miR-34a mimics, but also favouring the accumulation in the tumor vessels by the so-called enhanced permeability and retention effect [47].

In a previous report, we investigated the anti-MM activity of miR-34a mimics using a different lipidic emulsion [27] based on unknown patented composition. Here we proposed to use well characterized delivery system, that, for different application is presently used in clinical phase III trials for the delivery of siRNA (http://www.tekmirapharm.com, http://www.alnylam.com/index.php). Therefore, we have preferred to use SNALPs in the present manuscript because they are well known and characterized delivery systems, thus suitable for in vivo clinical translational studies. SNALPs have been used in different animal models of cancer for siRNA delivery [48], [49]. However, at our knowledge, the present study is the first which demonstrates the effectiveness of SNALPs for miRNA mimics systemic delivery in tumor xenograft.

In conclusion, in the present report, we provided novel information on miR-34a as a negative regulator of MM cell growth and we demonstrated that miRNA mimics are efficiently delivered *in vivo* by SNALP particles. Notably, we found a survival advantage for mice treated with miR-34a-containing SNALPs. Moreover, our data suggest that the anti-MM effects induced by miRNA-containing SNALPs were indeed due to the specific replacement of miR-34a in MM cells. Two points support this conclusions: i) no significant effects were induced by SNALP encapsulating a scramble sequence; ii) the anti-MM effects induced by SNALP miR-34a were paralleled by increased intratumor levels of miR-34a and decreased levels of its canonic target NOTCH1. Finally, the treatment did not produce evident toxicity since no changes in the mice weight and no detectable effects in some relevant organs (liver and kidney), where SNALPs are predicted to accumulate, were recorded at the end of treatment. All together these findings lay the groundwork for future translation of SNALP miR-34a in clinical setting.
